# SEA-MAKE score as a tool for predicting major adverse kidney events in critically ill patients with acute kidney injury: results from the SEA-AKI study

**DOI:** 10.1186/s13613-020-00657-9

**Published:** 2020-04-16

**Authors:** Theerapon Sukmark, Nuttha Lumlertgul, Kearkiat Praditpornsilpa, Kriang Tungsanga, Somchai Eiam-Ong, Nattachai Srisawat

**Affiliations:** 1 Thungsong Hospital, Nakhon Si Thammarat, Thailand; 2 Division of Nephrology, Department of Medicine, Faculty of Medicine, Chulalongkorn University, and King Chulalongkorn Memorial Hospital, Bangkok, 10330 Thailand; 3 Excellence Center for Critical Care Nephrology, King Chulalongkorn Memorial Hospital, Bangkok, Thailand; 4 Critical Care Nephrology Research Unit, Faculty of Medicine, Chulalongkorn University, Bangkok, Thailand; 5 Academic of Science, Royal Society of Thailand, Bangkok, Thailand; 6 Tropical Medicine Cluster, Chulalongkorn University, Bangkok, Thailand; 7 Center for Critical Care Nephrology; The CRISMA Center, Department of Critical Care Medicine, University of Pittsburgh School of Medicine, Pittsburgh, PA USA

**Keywords:** Major adverse kidney events (MAKE), Acute kidney injury (AKI), Clinical Predictive Score

## Abstract

**Background:**

Acute kidney injury (AKI) is a common problem in critically ill patients and associated with high rates of morbidity and mortality. Recently, Major Adverse Kidney Events (MAKE) were introduced as important kidney endpoints. If these endpoints can be predicted, then it may help the physicians to identify high-risk patients and provide the opportunity to have targeted preventive therapy. The objective of this study was to create a simplified scoring system to predict MAKE within 28 days among AKI patients in ICU.

**Methods:**

This is a prospective web-based multicenter cohort study that was conducted in adults who were admitted to the ICU in 17 centers across Thailand from 2013 to 2015. A predicting score was derived from the regression equation with Receiver Operating Characteristic (ROC) analysis to evaluate the diagnostic test and produce predictive models. Internal validation was obtained using the bootstrapping method.

**Results:**

From 5071 cases, 2856 (56%) had AKI. Among those with AKI, 1749 (61%) had MAKE. Among those that have MAKE, there were 1175 (41.4%) deaths, 414 (14.4%) were on dialysis and 1154 (40.7%) had non-recovery renal function. The simplified score points of low Glasgow coma scale was 3, tachypnea was 1, vasopressor use was 1, on mechanical ventilation was 2, oliguria was 2, serum creatinine rising ≥ 3 times was 5, high blood urea nitrogen was 3, low hematocrit was 2, and thrombocytopenia was 1. The area under ROC curve for optimism corrected performance was 0.80 (0.78, 0.81). When the cut-off value was 7, the sensitivity, specificity, positive likelihood ratio, and positive predictive values were 0.75, 0.76, 3.10, and 0.84, respectively. When the scoring system was calibrated, the α intercept and β slope were 1.001 and 0, respectively.

**Conclusions:**

SEA-MAKE scoring system is a new simplified clinical tool that can be used to predict major adverse kidney events in AKI patients. The simplicity of the scoring system is highly likely to be used in resource-limited settings. However, external validation is necessary before widespread use.

## Background

Acute kidney injury (AKI) frequently occurs up to 70% in critically ill patients; the morbidity and mortality rate among patients with AKI are high [1]. Patients who have survived from AKI will be exposed to long-term risks of progression to chronic kidney disease (CKD) and end-stage renal disease (ESRD) which pose a heavy burden on the healthcare system. Even though experimental models have yielded many promising agents for AKI treatment, however, these could not be translated into clinical practice [2, 3].

To address these issues, the National Institute of Diabetes and Digestive and Kidney Disease (NIDDK) workgroup for Clinical Trials in Acute Kidney Injury recommended to use “a composite endpoint of death, provision of dialysis, and sustained loss of kidney function” for phase 3 clinical trials in established AKI [4] which is commonly referred to as Major Adverse Kidney Events (MAKE), analogous to the Major Adverse Cardiac Events (MACEs) for coronary artery diseases [5]. MAKE comprises three clinically important outcomes resulting in reasonable event rates and simultaneously reflects the short-term and long-term patient-centered outcomes [6].

Several retrospective studies used electronic medical records to design prediction models for MAKE [7–9]. However, there are no prediction models for MAKE based on data from a prospective study. If MAKE can be predicted during hospitalization, it can help identify high-risk AKI patients for possible targeted interventions.

The objective of this study was to create a scoring system to predict major adverse kidney events within 28 days (MAKE 28) after AKI in ICU patients that is easy to use and applicable to many hospitals, especially in resource-limited settings.

## Methods

### Patients and study design

This was a prospective multicenter observational cohort study or also known as the Southeast Asia AKI cohort study that is conducted in 17 ICUs at 16 hospitals from various regions across Thailand. The study sites were composed of university, regional, and provincial hospitals. All patients aged older than 15 years who were admitted to the participating ICUs from February 2013 to July 2015 were enrolled into the study. Patients with ESRD on chronic dialysis were excluded. Only data from the first admission were used if the patients have been admitted to the hospital several times during the study period. Formal sample size was not calculated because it was fixed to the Southeast Asia AKI cohort [10]. All available data were used to maximize the power and generalizability of the proposed model.

### Ethics statement

The study protocol was reviewed by the Institutional Review Board at each participating site and the informed consent was waived.

### Data collection

All data were collected in a web-based platform after the patient was registered online at the study sites. Demographic, clinical and laboratory data were collected. The following demographic data were collected: age, body mass index, sex, co-morbidity diseases (hypertension, diabetes, coronary artery, cerebrovascular disease, malignancy, and chronic kidney disease), timing of hospital and ICU admission, and the patient’s principle diagnosis at ICU admission. Clinical data at ICU admission were collected: the patient’s vital signs, fluid balance status, whether or not the patient used vasopressor or mechanical ventilation, renal replacement therapy, Acute Physiology and Chronic Health Evaluation (APACHE) II score, and Sequential Organ Failure Assessment (SOFA) scores for the first 3 days after being admitted to the hospital. Laboratory data such as complete blood count, serum creatinine, and blood urea nitrogen levels, if available, were obtained. The data were collected every day for the first 7 days and then weekly until day 28. However, only parameters from the first day were used as predictors to create the model.

### Study outcomes

The primary outcome was patients who met one or more criteria for Major Adverse Kidney Events within 28 days (MAKE28): death, provision of dialysis, and/or sustained loss of the kidney function. Hospital death was defined as death from any cause before hospital discharge within 28 days after ICU admission. Provision of dialysis was defined as receipt of any modality of renal replacement therapy before hospital discharge within 28 days after ICU admission. Sustained loss of kidney function was defined as a final serum creatinine value before hospital discharge within 28 days after ICU admission that was 200% or more than the baseline creatinine value.

### Study definitions

AKI was diagnosed by KDIGO criteria [3]. For the baseline serum creatinine, the most recent available value before hospital admission, within 1 year, was used. If the true baseline creatinine level was not available and the patients had no previous history of CKD, we estimated the baseline serum creatinine using the lowest value between the serum creatinine value at the time of hospital admission and the back calculation of serum creatinine from the Modification of Diet in Renal Disease (MDRD) equation using a glomerular filtration rate of 75 mL/min/1.73 m^2^ [11] as recommended in the KDIGO guideline [3]. For the urine output criteria, it was modified to using 24-h urine output. The patients who had urine output > 0.5 mL/kg/h were defined as no AKI, 0.3–0.5 mL/kg/h were defined as KDIGO stage 2, and < 0.3 mL/kg/h were defined as KDIGO stage 3 [11]. MAKE was defined as a composite endpoint of death, provision of dialysis, and sustained loss of kidney function [4].

The International Classification of Disease, 10th Revision coding was used to classify the diagnosis of ICU admission. The primary diagnosis for ICU admission and etiologies of AKI were determined by the study team. The definition of “renal-related disease” was AKI, glomerular disease, electrolyte imbalance related to renal disorders and/or stones in the urinary tract system. Hypertension was defined according to the seventh Report of the Joint National Committee on Prevention, Detection, Evaluation, and Treatment of High Blood Pressure (JNC 7) [12]. Chronic kidney disease was diagnosed according to KDIGO 2012 Clinical Practice Guideline for the Evaluation and Management of Chronic Kidney Disease [13].

The actual body weight value before hospital admission was used to calculate the rate of urine flow, if unavailable, the ideal body weight was calculated from the following formula: height (cm)—100 for male and height (cm)—110 for female. Body mass index (BMI) class was defined using the World Health Organization Expert Consultation criteria: optimal BMI was 18.5–22.9 kg/m^2^, overweight had BMI > 23 kg/m^2^, and obesity had BMI > 27.5 kg/m^2^ [14]. Almost all of the patients in this study were Asian.

### Statistical analyses

Categorical data were presented as counts and percentages. Continuous data were presented as mean and standard deviation (SD), if normally distributed, or median with interquartile range, if non-normally -distributed. Comparison between MAKE and non-MAKE groups was performed using a Pearson’s Chi Square asymptotic test and a Fisher’s exact test for categorical variables. Meanwhile, a Student’s *t* test (if data were normally distributed) or Kruskal–Wallis one-way analysis of variance by ranks (if data were non-normally distributed) was performed for continuous variables. The normality was assessed by graph visualization, and the similarity between the mean and the median was compared. Statistical analyses were done using IBM SPSS Statistics version 21. *P* value < 0.05 was considered to be statistically significant.

In the development cohort, all potential clinical factors were initially considered. Variables that were statistically significant in the univariate logistic regression method were inputted into the multivariate logistic regression method. In the multivariable analysis models, each clinical factor was adjusted for age, sex, body mass index, and co-morbidities such as hypertension, diabetes, coronary artery disease, cerebrovascular disease, malignancy, and chronic kidney disease to examine the potential predictability. Variables that were statistically significant in the multivariate logistic regression method were selected as predictors of MAKE. Then, significant predictors were included in the final analysis. Candidate variables with more than 25% missing data were eventually excluded. Data that were not missing completely at random, multiple imputations (*n* = 10) for the missing candidate variables were performed in the final model. Multivariable binary logistic regression analysis with adjustment for co-variates was operated under stepwise Forward Wald selection method. Parameter estimate values were obtained from pooled data of 10 imputations. The full model was converted to a simplified score points using Schneeweiss’s scoring system [15]. The variance inflation factors (VIFs) and tolerance coefficients were computed to test multicollinearity among the co-variates. Value of VIF exceeding 10 are often regarded as indicating multicollinearity, but in weaker models, which is often the case in logistic regression, value above 2.5 may be a cause for concern [16]. A tolerance value below 0.1 indicated that there was a serious collinearity problem and if the value was less than 0.2, this indicated that there was a potential collinearity problem [17, 18].

Internal validation was performed using bootstrapping method [19]. A total of 2000 bootstrap samples were drawn, and the final model containing all included variables was fitted in each bootstrap sample. This was followed by re-simplification of the score and point assignment using the Schneeweiss’s scoring system [15]. The apparent performance of the score was evaluated using the following performance measures: Nagelkerke’s *R*^2^ (overall performance) [20], area under the receiver operating characteristic curve (AUC) (able to differentiate between MAKE and non-MAKE patients), and a calibration plot [19, 21]. If AUC = 1, then this indicated that there was a perfect discrimination. If the AUC = 0.5, then the discrimination was no better than chance. Calibration intercept α and slope β from the linear regression of the observed outcomes (dependent variable) versus the predicted risks (independent variable) were acquired to show that the predictions were on average, close to the average observed outcome. In the calibration curve analysis, the intercept *α* = 0 and slope *β* = 1 implied that the models were well-calibrated.

We also tried to compare the discrimination (AUC) of the SEA-MAKE score to APACHE II, SOFA day 1, and SOFA-Kidney day 1 in this internal validation analysis even though we relegalized that the limitation of using internal validation might overestimate the performance of SEA-MAKE score over the other score and the comparison should be operated under a true external validation circumstance.

## Results

**Fig. 1:**
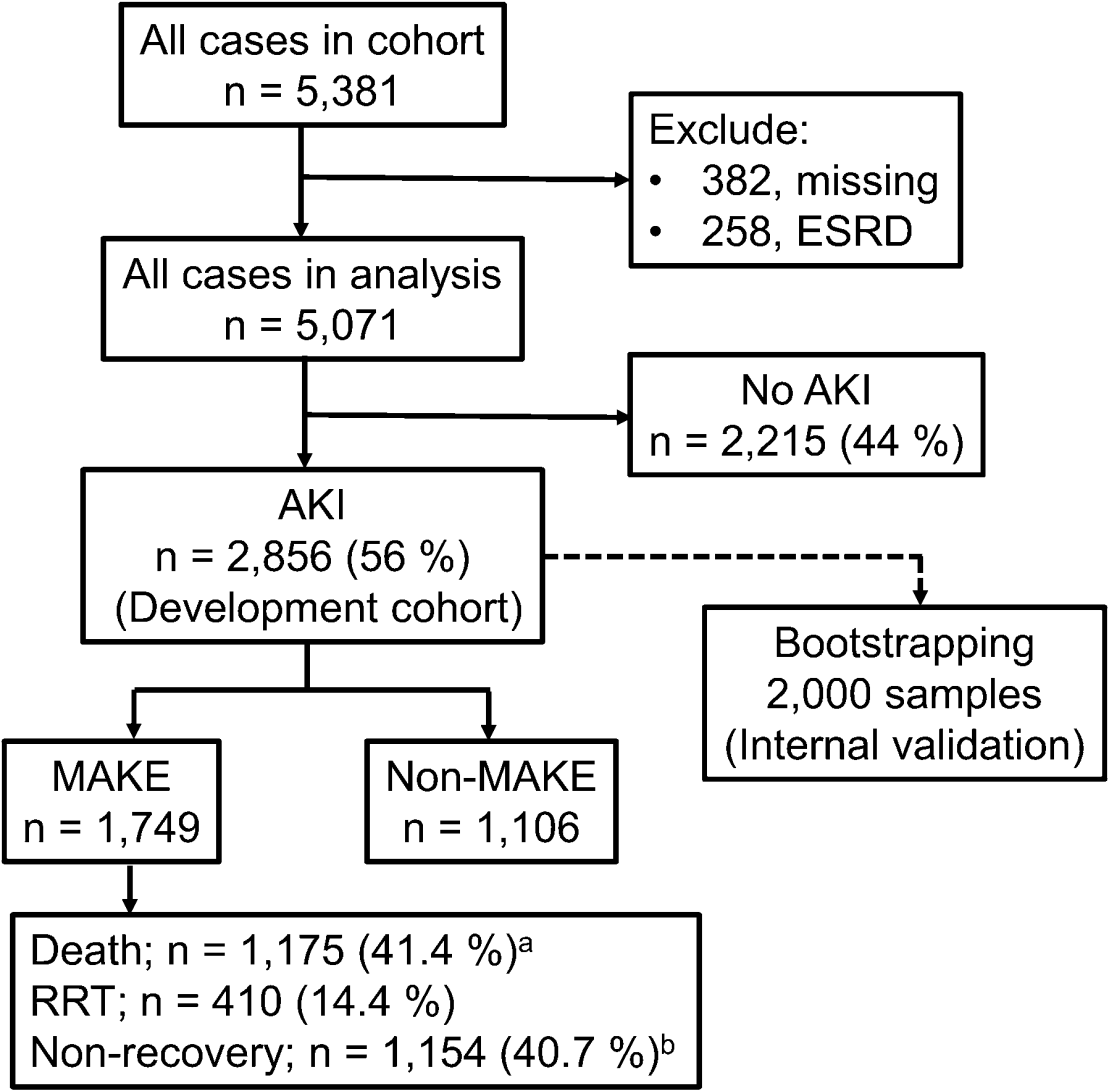
Flow chart of the study. ^a^Death rate; 15 cases had missing data, ^b^Non-recovery; 23 cases had missing data

All 5381 cases were eligible for this cohort. We excluded 382 cases because they did not have blood and urine samples, and 258 cases had ESRD. After excluding those patients, we had 5071 cases that were available for analysis (Fig. 1). Of the analyzable cases, 2856 (56%) were AKI patients and 2215 (44%) were non-AKI patients. Among AKI patients, 1749 participants met one or more criteria of MAKE: death (*n* = 1175; 41.4%), new RRT (*n* = 410; 14.4%), and renal non-recovery (*n* = 1154; 40.7%). All cases of AKI were included in the development cohort to create the clinical score (SEA-MAKE score) for predicting MAKE28. Majority of the principle diagnoses upon ICU admission in the development cohort had pulmonary diseases (29.7%), cardiovascular diseases (26.5%), and infectious diseases (15.8%) (Additional file 1: Table S1).

### Development cohort analysis

In the development cohort, univariate analysis for comparisons of clinical and laboratory characteristics between MAKE and non-MAKE at the time of enrollment is shown in Table 1. Many parameters such as diabetes, CKD, Glasgow Coma scale, body temperature, heart rate, respiratory rate, mean arterial pressure, hematocrit, platelet count, serum sodium, serum potassium, blood urea nitrogen, serum creatinine, total bilirubin, PaO_2_/FiO_2_ ratio, arterial pH, urine output, net fluid balance, mechanical ventilation, and vasopressor use were found to be significantly different between MAKE and non-MAKE patients. However, age, body mass index, sex, hypertension, coronary artery disease, cerebrovascular disease, malignancy and chronic health points were not found to be significantly different between the two groups.

**Table 1:** Patients’ clinical and laboratory characteristics between MAKE and Non-MAKE patients in the Development Cohort (*n* = 2856)

Clinical characteristics	MAKE (*n* = 1749)	Non-MAKE (*n* = 1106)	*P* value	Missing values, *n* (%)
Age, years	65.8 ± 20.0	65.2 ± 16.7	0.460	0
BMI, kg/m^2^	23.50 ± 15.13	23.64 ± 5.89	0.767	4 (0.1)
Sex Male, *n* (%)	944 (54.0)	634 (57.3)	0.082	1 (0)
Co-morbidity diseases				
HT	784 (44.8)	494 (44.7)	0.969	2 (0.1)
DM	524 (30.0)	281 (25.5)	0.009	11 (0.4)
CAD	183 (10.5)	117 (10.6)	0.950	10 (0.4)
Cerebrovascular disease	119 (6.8)	69 (6.3)	0.588	9 (0.3)
Malignancy	143 (8.2)	82 (7.4)	0.477	10 (0.4)
CKD	278 (15.9)	93 (8.4)	< 0.001	1 (0)
Chronic health points			0.148	1 (0)
0	1326 (75.8)	818 (74.0)		
2	41 (2.3)	39 (3.5)		
5	382 (21.8)	249 (22.5)		
Glasgow Coma Scale	9.18 3.73	11.49 2.97	< 0.001	0
Temperature, degree Celsius			< 0.001	1 (0)
≤ 29.9	2 (0.1)	0		
32–33.9	17(1.0)	7 (0.6)		
34–35.9	176 (10.1)	70 (6.3)		
36–38.4	1222 (69.9)	875 (79.1)		
38.5–38.9	146 (8.3)	82 (7.4)		
39–40.9	175 (10.0)	71 (6.4)		
≥ 41	11 (0.6)	1 (0.1)		
Heart rate, beats per minute			< 0.001	1 (0)
≤ 39	9 (0.5)	9 (0.8)		
40–54	26 (1.5)	19 (1.7)		
55–69	81 (4.6)	92 (8.3)		
70–109	737 (42.1)	563(50.9)		
110–139	672 (38.4)	324 (29.3)		
140–179	216 (12.3)	98 (8.9)		
≥ 180	8 (0.5)	1 (0.1)		
Respiratory rate, breaths per minute			< 0.001	1 (0)
≤ 5	1 (0.1)	1 (0.1)		
6–9	1 (0.1)	3 (0.3)		
10–11	6 (0.3)	6 (0.5)		
12–24	890 (50.9)	760 (68.7)		
25–34	689 (39.4)	292 (26.4)		
35–49	161 (9.2)	44 (4.0)		
≥ 50	1 (0.1)	0		
Mean arterial pressure, mmHg			< 0.001	5 (0.2)
≥ 70	734 (42.0)	678 (61.4)		
< 70	81 (4.6)	78 (7.1)		
Dopamine ≤ 5 or any	60 (3.4)	39 (3.5)		
Dopamine > 5–15 or norepinephrine ≤ 0.1	150 (8.6)	97 (8.8)		
4, Dopamine > 15 or norepinephrine > 0.1	722 (41.3)	212 (19.2)		
Hematocrit, %			< 0.001	1 (0)
< 20	124 (7.1)	34 (3.1)		
20–29.9	666 (38.1)	244 (22.1)		
30–45.9	882 (50.4)	748 (67.6)		
46–49.9	47 (2.7)	58 (5.2)		
50–59.9	24 (1.4)	20 (1.8)		
≥ 60	6 (0.3)	2 (0.2)		
Platelet count, per µL			< 0.001	3 (0.1)
≥ 150,000	1057 (60.5)	845 (76.5)		
< 150,000	265 (15.2)	136 (12.3)		
< 100,000	238 (13.6)	76 (6.9)		
< 50,000	124 (7.1)	36 (3.3)		
< 20,000	64 (3.7)	12 (1.1)		
Serum sodium, mEq/L			< 0.001	1 (0)
≤ 110	2 (0.1)	1 (0.1)		
111–119	15 (0.9)	9 (0.8)		
120–129	184 (10.5)	75 (6.8)		
130–149	1469 (84.0)	998 (90.2)		
150–154	64 (3.7)	15 (1.4)		
155–159	10 (0.6)	5 (0.5)		
160–179	4 (0.2)	3 (0.3)		
≥ 180	1 (0.1)	0		
Serum potassium, mEq/L			< 0.001	1 (0)
< 2.5	18 (1.0)	15 (1.4)		
2.5–2.9	81 (4.6)	39 (3.5)		
3–3.4	261 (14.9)	183 (16.5)		
3.5–5.4	1198 (68.5)	823 (74.4)		
5.6–5.9	87 (5.0)	28 (2.5)		
6–6.9	69 (3.9)	13 (1.2)		
≥ 7	35 (2.0)	5 (0.5)		
Blood urea nitrogen, mg/dL	39.8 (23.9, 65.0)	23 (15.0, 35.0)	< 0.001^a^	1 (0)
Serum creatinine, mg/dL			< 0.001	1 (0)
< 0.6	65 (3.7)	73 (6.6)		
0.6–1.4	489 (28.0)	587 (53.1)		
1.5–1.9	264 (15.1)	213 (19.3)		
2–3.4	453 (25.9)	165 (14.9)		
≥ 3.5	478 (27.3)	68 (6.1)		
Total bilirubin, mg/dL			< 0.001	8 (0.3)
< 1.2	1266 (72.6)	911 (82.6)		
1.2–1.9	151 (8.7)	82 (7.4)		
2.0–5.9	211 (12.1)	87 (7.9)		
6.0–11.9	78 (4.5)	16 (1.5)		
≥ 12	39 (2.2)	7 (0.6)		
PaO_2_/FiO_2_			< 0.001	2 (0.1)
> 400	368 (21.0)	296 (26.8)		
≤ 400	332 (19.0)	250 (22.6)		
≤ 300	494 (28.2)	316 (28.6)		
≤ 200 with respiratory support	335 (19.2)	181 (16.4)		
≤ 100 with respiratory support	220 (12.6)	62 (5.6)		
Arterial pH			< 0.001	1 (0)
7.15	154 (8.8)	15 (1.4)		
7.15–7.24	157 (9.0)	32 (2.9)		
7.25–7.32	205 (11.7)	83 (7.5)		
7.33–7.49	875 (50.0)	689 (62.3)		
7.5–7.59	106 (6.1)	86 (7.9)		
7.6–7.69	72 (4.1)	55 (5.0)		
≥ 7.7	73 (4,2)	27 (2.4)		
Urine output, liter	0.59 (0.19, 1.31)	0.86 (0.4, 1.56)	< 0.001^a^	23 (0.8)
Net fluid balance, liter	1.31 (0.36, 2.63)	0.70 (0, 1.62)	< 0.001^a^	22 (0.8)
Mechanical ventilation (day 1)	1541 (88.3)	772 (69.9)	< 0.001	5 (0.2)
Vasopressor (day 1)	939 (53.8)	350 (31.7)	< 0.001	6 (0.2)
APACHE II	21.85 ± 6.82	15.89 ± 6.53	< 0.001	0
SOFA day 1	9.40 ± 3.98	5.83 ± 3.50	< 0.001	6 (0.2)
LOS in ICU	5 (2, 10)	4 (2, 8)	0.243^a^	18 (0.6)
LOS in hospital	11 (5, 24)	13 (7, 24)	0.002^a^	7 (0.2)

^a^Independent Samples Kruskal–Wallis Test

**Fig. 2:**
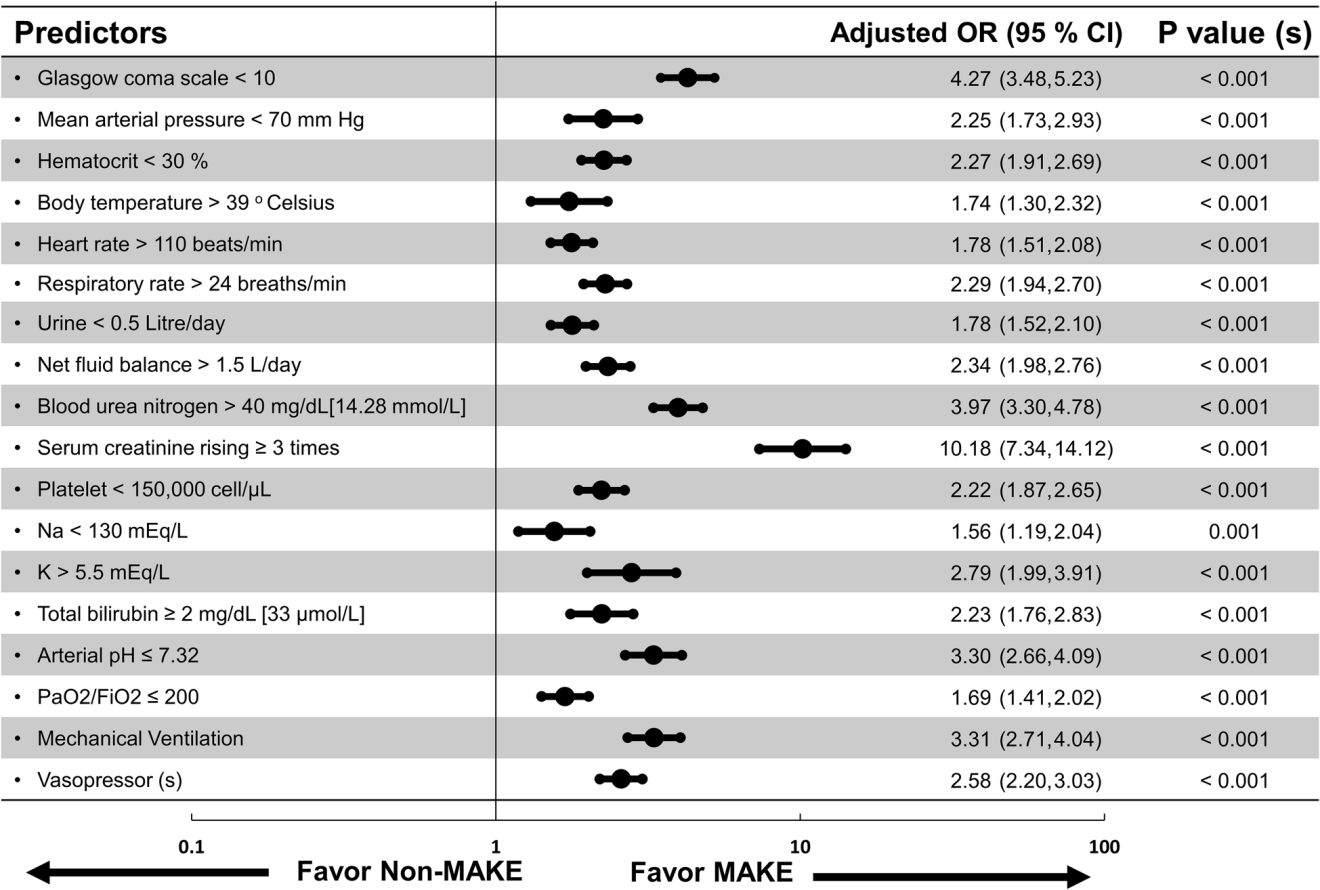
Forest plot in log-10 scale of odds ratio (adjusted for age, sex, body mass index and co-morbidities such as hypertension, diabetes, coronary artery disease, cerebrovascular disease, malignancy, and chronic kidney disease) in predicting Major Adverse Kidney Events (MAKE28)

In the logistic regression analysis, adjusted for age, sex, body mass index and co-morbidity (hypertension, diabetes coronary artery disease, cerebrovascular disease, malignancy and chronic kidney disease), it was determined that MAKE had a significantly lower Glasgow coma scale [OR (95% CI) 4.27 (3.48, 5.23)], lower mean arterial pressure [OR (95% CI) 2.25 (1.73, 2.93)], lower hematocrit [OR (95% CI) 2.27 (1.91, 2.69)], lower urine output [OR (95% CI) 1.78 (1.52, 2.10)], and lower platelet count [OR (95% CI) 2.22 (1.87, 2.65)]. Conversely, it was determined that MAKE had a significantly higher body temperature [OR (95% CI) 1.74 (1.30, 2.32)], higher heart rate [OR (95% CI) 0.78 (1.51, 2.08)], higher respiratory rate [OR (95% CI) 2.29 (1.94, 2.70)], higher positive fluid balance [OR (95% CI) 2.34 (1.98, 2.76)], higher blood urea nitrogen [OR (95% CI) 3.97 (3.30, 4.78)], and especially higher proportion of serum creatinine rising ≥ 3 times from the baseline level [OR (95% CI) 10.18 (7.34, 14.12)] (Fig. 2).

In creating the SEA-MAKE score using multivariable regression-based method, after multiple imputations were performed for the missing values, the candidate predictor variables were included in the analysis under the Forward Wald selection with adjustment for the co-variates. Nine predictors were significant in the final model: low Glasgow coma scale [OR (95% CI) 2.72 (2.17, 3.40)], tachypnea [OR (95% CI) 1.55 (1.29, 1.86)], vasopressor use [OR (95% CI) 1.56 (1.30, 1.88)], on mechanical ventilation [OR (95% CI) 1.81 (1.45, 2.27)], oliguria [OR (95% CI) 1.78 (1.48, 2.14)], serum creatinine rising ≥ 3 times [OR (95% CI) 4.77 (3.38, 6.73)], high blood urea nitrogen [OR (95% CI) 2.33 (1.89, 2.86)], low hematocrit [OR (95% CI) 1.81 (1.50, 2.19)], and thrombocytopenia [OR (95% CI) 1.40 (1.15, 1.70)] (Table 2). The simplified score points (Schneeweiss’s scoring system) of low Glasgow coma scale was 3, tachypnea was 1, vasopressor use was 1, on mechanical ventilation was 2, oliguria was 2, serum creatinine rising ≥ 3 times was 5, high blood urea nitrogen was 3, low hematocrit was 2, and thrombocytopenia was 1. All of the variance inflation factors (VIFs) were less than 1.3 and the tolerance coefficients exceeded 0.79, meaning that there was no potential multicollinearity present among the co-variates.

**Table 2:** Creating the simplified SEA-MAKE scoring system using the multivariable regression method^a^

Predictors	B	S.E.	Adjusted odds ratio^b^ (95% CI)	P	Simplified scores^c^
Glasgow coma scale < 10	1.00	0.11	2.72 (2.17, 3.40)	< 0.001	3
Respiratory rate > 24 breaths/min	0.44	0.09	1.55 (1.29, 1.86)	< 0.001	1
Vasopressor use	0.45	0.09	1.56 (1.30, 1.88)	< 0.001	1
On mechanical ventilation	0.60	0.11	1.81 (1.45, 2.27)	< 0.001	2
Urine output < 0.5 L/day	0.58	0.09	1.78 (1.48, 2.14)	< 0.001	2
Serum creatinine rising ≥ 3 times	1.56	0.18	4.77 (3.38, 6.73)	< 0.001	5
Blood urea nitrogen ≥ 40 mg/dL [14.28 mmol/L]	0.84	0.11	2.33 (1.89, 2.86)	< 0.001	3
Hematocrit < 30%	0.59	0.10	1.81 (1.50, 2.19)	< 0.001	2
Platelet counts < 150 × 10^3^ cell/µL	0.34	0.10	1.40 (1.15, 1.70)	0.001	1

^a^Pooled data from 10 imputations after Forward Wald selection method in binary logistic regression model

^b^Adjusted for co-variates

^c^Schneeweiss’s scoring system

All of the variance inflation factors (VIFs) coefficients in collinearity statistics were less than 1.3 and tolerance coefficients exceeded 0.79 in multicollinearity test among all co-variates

### Score performance and internal validation

**Fig. 3:**
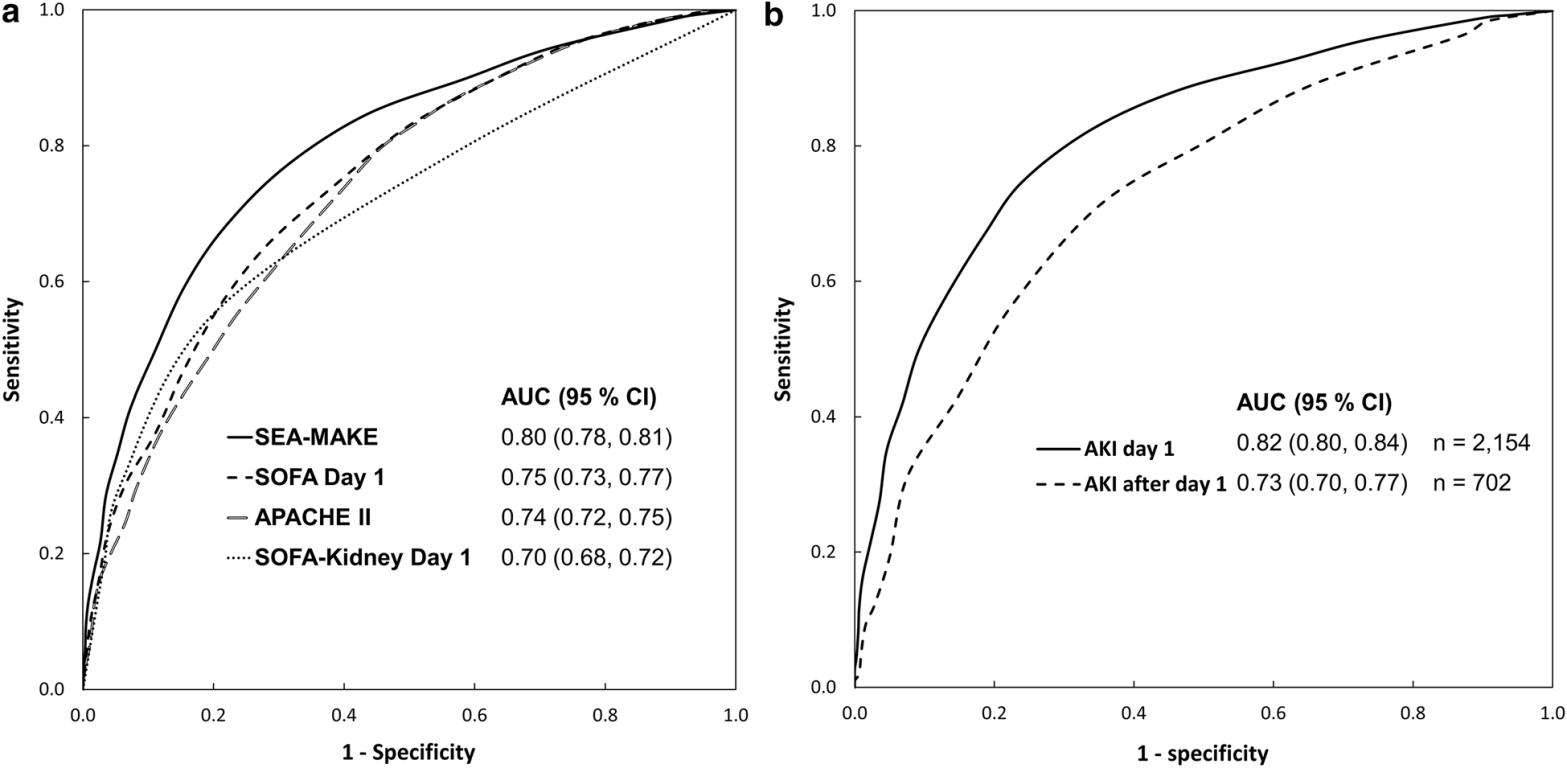
The area under the receiver operating characteristic (ROC) curve to assess the performance of the scoring system to predict MAKE. **a** The performance of SEA-MAKE was compared to SOFA Day 1, APACHEI II, and SOFA-Kidney Day1. **b** The ability of SEA-MAKE scoring system in distinguishing between the patients with AKI on the first day (AKI day 1) and after the first day (AKI after day 1)

The overall performance of the score (Nagelkerke’s *R*^2^) was 0.329. Discrimination (AUC) of apparent performance was 0.797 (95% CI 0.780–0.813), bias-corrected bootstrap performance was 0.799 (95% CI 0.780–0.813) (Additional file 1: Figure S1), and optimism-corrected performance was 0.795. When we compared the discrimination of SEA-MAKE score to SOFA day 1, APACHE II, and SOFA kidney day 1, the AUC (95% CI) of those scores were 0.75 (0.73, 0.77), 0.74 (0.72, 0.75) and 0.70 (0.68, 0.72), respectively (Fig. 3a). We also found that SEA-MAKE score had higher discriminatory power when applied to patients with AKI on the first day (*n* = 2154) compared to after the first day (*n* = 702) with AUC (95%) of 0.82 (0.80, 0.84) and 0.73 (0.70, 0.77), respectively (Fig. 3b). Based on the simplified model, SEA-MAKE score can be calculated by adding the simplified score point of each predictor. When the cut-off value was 7 for AKI in the first day group (AKI D1), the sensitivity was 0.75, specificity was 0.76, positive likelihood ratio was 3.10, and the positive predictive value was 0.84. When the cut-off value was 5 in AKI after the first day group (AKI after D1), the sensitivity was 0.71, specificity was 0.65, positive likelihood ratio was 2.03, and the positive predictive value was 0.71 (Additional file 1: Table S2).

In the calibration curve analyses, the X axis represented the predicted risk for MAKE, the Y axis represented the observed risk for MAKE, the blue straight line represented a perfect fit, and the red dots represented the average MAKE of bias-corrected bootstrap samples in each bins. The calibration intercept *α* = 0 and calibration slope *β* = 1.001 are shown in Additional file 1: Figure S2.

## Discussion

Although many AKI risk prediction models to predict AKI in critically ill patients have been extensively studied and developed, yet MAKE prediction model still has not been proposed [22–24]. SEA-MAKE score is the first simplified clinical score for predicting major adverse kidney events after AKI in ICU patients that can be used as the point of care (POC). In summary, the nine parameters that were used to develop the score were Glasgow coma scale, respiratory rate, vasopressor use, on mechanical ventilation, urine output, serum creatinine rising, blood urea nitrogen, hematocrit, and platelet count. If multiple laboratory values of each predictor were present, the worst value of each predictor measured within first 24 h after admission to the ICU should be used. Importantly, the score is easy to calculate and can be applied to most hospitals, even in resource-limited settings. Even though the MAKE predicting model was first published by McKown et al. in 2017 [9], that model was developed from a retrospective study using the electronic health record to create an exponential equation-based model. Also, the previous developed scoring system was difficult to use in real-life patient care setting. However, the SEA-MAKE score can be used as POC (Additional file 1: Figure S3). The SEA-MAKE scoring system can be used to assess MAKE in the first 24 h before the patients develop AKI or at 48 h for patients who develop AKI-after-the-first-day (AKI after D1) (Additional file 1: Table S3). We also found that predictive ability of SEA-MAKE score was higher in university and regional hospitals than provincial hospitals (Additional file 1: Table S4).

Formal sample size calculation was not calculated in our study because it was fixed to the Southeast Asia AKI study [10]. All available data of the cohort were used to maximize the power and generalizability. However, it is important to have an adequate sample size when developing a prediction model; it is the standard method to determine what counts as “adequate” or not [25]. Some experts may suggest adequate sample size of least 10 events per candidate variable for the derivation of a model and at least 100 events for validation studies [26]. Based on this concept, this study population was, by far, larger than the minimal requirement. We excluded patients who lacked blood and urine samples because we could not identify who had AKI or not. In addition, the parameters used to predict MAKE were mostly related to blood and urine data. Thus, without the blood and urine data, we will not be able to use the scoring system to predict which AKI patient will develop MAKE.

Regarding the use of composite endpoints in this study, there were several advantages. First, the composite endpoint increases the event rates which, in turn, enhance statistical power. Second, composite endpoints may reduce bias due to competing risk (e.g., death is a competing risk for incident AKI whereas RRT is a competing risk for doubling of serum creatinine level) [6]. However, the disadvantages of using composite endpoint also need to be addressed. First, we did not know which predictors contributed to each component of the composite outcomes. Second, it may be misleading when outcomes vary significantly in their clinical relevance and importance to the patients, for example, when we have the least important outcomes contributing to the greatest number of events [6]. So, we also investigated the AUC of SEA-MAKE score among the composite endpoints of MAKE in each separated endpoint as shown in Additional file 1: Table S5.

There are several strengths in this study. First, this was a large multicenter, systematically prospectively collected-data study, conducted in all regions across Thailand. All of the three levels of health service hospitals (university hospital, regional hospital, and provincial hospital) were included in this study. Second, the patients who were enrolled in this cohort had been admitted with various types of diseases that is similar to real-life situation. Third, as previously mentioned, this study was probably the first to develop a simplified score that can be practically used as POC even in resource-limited settings. Fourth, multicollinearity was not found among the nine predictors which were tested by variance inflation factors and tolerance. Fifth, we used the regression coefficient-based scoring system rather than risk ratio-based scoring system. This was based on the reason that the regression coefficient-based scoring system provided better mathematic correction, and better performance in fitting the data than risk ratio-based scoring system [27].

However, several limitations need to be discussed. First, the diversity of the patients in the study population could be problematic because specific diseases, especially the cause of AKI, may affect clinical courses and outcomes but we could not include it in this score. Second, when we compared the performance of SEA-MAKE score to APACHE II, and SOFA (Fig. 3a), we found that the internal validation was suboptimal because it was not a true external validation. In addition, APACHE II, SOFA day 1, and renal SOFA day 1 were not directly designed to predict MAKE. Therefore, this result needs to be interpreted with caution. Third, MAKE28 outcome of this study was only based on the in-hospital data. Hence, our MAKE28 could not be extrapolated to the events after hospital discharge.

## Conclusions

The SEA-MAKE score is probably the first simplified clinical scoring system to predict MAKE 28 in critically ill patients with AKI. This tool may be useful in helping clinicians predict MAKE in routine ICU care. The tool is easy-to use and feasible even in resource-limited settings. The model performance was adequate when internally validated. However, external validation is necessary before widespread use.

## Supplementary information

**Additional file 1. Table S1.** Distribution of the diseases diagnosed in the development cohort (*n* = 2,856). **Table S2.** Performance of SEA-MAKE score at various cut-off values to predict major adverse kidney events within 28 days (MAKE28). **Table S3.** Discrimination comparison of SEA-MAKE score in patients with AKI-after-the-first-day (AKI after D1) assessed at 24, 48, and 72 h after hospital admission (*n* = 702). **Table S4.** Predictive ability of SEA-MAKE score in different settings. **Table S5.** Discrimination of SEA-MAKE score for composite MAKE and each separated endpoints within 28 days after AKI. **Figure S1.** The area under the receiver operating characteristic (ROC) curve for MAKE prediction of the apparent performance and bias-corrected bootstrap performance. **Figure S2.** Calibration curve analysis comparing between observed and predicted risk of MAKE at each cut-off point of SEA-MAKE score during internal validation. **Figure S3.** The proposed diagram of SEA-MAKE score in clinical practice as point of care. **Table S6.** TRIPOD checklist.

## Data Availability

If the request is reasonable, the corresponding author can share the data from this study to the requestor.

## Abbreviations

AKI: Acute Kidney Injury
APACHE: Acute Physiology and Chronic Health Evaluation
AUC: Area under ROC curve
BMI: Body mass index
CI: Confidence interval
ESRD: End-Stage Renal Disease
ICU: Intensive Care Unit
KDIGO: Kidney disease: Improving global outcomes
MAKE: Major Adverse Kidney Events
MDRD: Modification of Diet in Renal Disease
OR: Odds ratio
ROC: Receiver Operating Characteristic
RRT: Renal replacement therapy
SEA: Southeast Asia
SD: Standard deviation
SOFA: Sequential Organ Failure Assessment
VIF: Variance inflation factors
