# Supplementary material for: SEA-MAKE score as a tool for predicting major adverse kidney events in critically ill patients with acute kidney injury: results from the SEA-AKI study
Source: Ann Intensive Care. 2020 Apr 16;10:42. doi: 10.1186/s13613-020-00657-9 (PMC7162998; doi:10.1186/s13613-020-00657-9)
Supplement: Supplementary file 1 — Additional file 1. Table S1. Distribution of the diseases diagnosed in the development cohort (n = 2,856). Table S2. Performance of SEA-MAKE score at various cut-off values to predict major adverse kidney events within 28 days (MAKE28). Table S3. Discrimination comparison of SEA-MAKE score in patients with AKI-after-the-first-day (AKI after D1) assessed at 24, 48, and 72 h after hospital admission (n = 702). Table S4. Predictive ability of SEA-MAKE score in different settings. Table S5. Discrimination of SEA-MAKE score for composite MAKE and each separated endpoints within 28 days after AKI. Figure S1. The area under the receiver operating characteristic (ROC) curve for MAKE prediction of the apparent performance and bias-corrected bootstrap performance. Figure S2. Calibration curve analysis comparing between observed and predicted risk of MAKE at each cut-off point of SEA-MAKE score during internal validation. Figure S3. The proposed diagram of SEA-MAKE score in clinical practice as point of care. Table S6. TRIPOD checklist. [file 13613_2020_657_MOESM1_ESM.docx]

**Table of Contents**

| **Table S1** | **Distribution of disease system in principle diagnosis in development cohort (n=2,856)** |
| --- | --- |
| **Table S2** | **Performance of SEA-MAKE score at various cutoff values to predict major adverse kidney events within 28 days (MAKE28)** |
| **Table S3** | **Discrimination comparison of SEA-MAKE score in patients with AKI-after-the-first-day (AKI after D1) assessing at 24^th^, 48^th^, and 72^th^ hour of admission (n = 702)** |
| **Table S4** | **Predictive ability of SEA-MAKE score in different setting** |
| **Table S5** | **Discrimination of SEA-MAKE score for composite MAKE and each separated endpoints within 28 days after AKI** |
| **Figure S1** | **The area under the receiver operating characteristic (ROC) curve for MAKE prediction of the apparent performance and bias-corrected bootstrap performance** |
| **Figure S2** | **Calibration curve analysis comparing between observed and predicted risk of MAKE at each cut-point of SEA-MAKE score in internal validation** |
| **Figure S3** | **The proposed diagram of SEA-MAKE score in clinical practice at the point of care** |
| **Table S6** | **TRIPOD Checklist** |

**Table S1. Distribution of disease system in principle diagnosis in development cohort (n = 2,856)**

| **Principle diagnosis** | **Cases** | **%** |
| --- | --- | --- |
| - Pulmonary diseases | 848 | 29.7 |
| - Cardiovascular diseases | 757 | 26.5 |
| - Infectious diseases | 452 | 15.8 |
| - Gastrointestinal diseases | 212 | 7.4 |
| - Neurologic diseases | 141 | 4.9 |
| - Renal diseases | 123 | 4.3 |
| - Oncologic diseases | 100 | 3.5 |
| - Surgical diseases | 97 | 3.4 |
| - Metabolic and toxic diseases | 61 | 2.1 |
| - Hematologic diseases | 26 | 1.0 |
| - Rheumatologic diseases | 12 | 0.4 |

Missing data 27 cases

**Table S2. Performance of SEA-MAKE score at various cutoff values to predict major adverse kidney events within 28 days (MAKE28)**

|  | **Cutoff values** | **Sensitivity** | **Specificity** | **+ LR^!^** | **- LR**^!!^ | **PPV**^#^ | **NPV**^$^ |
| --- | --- | --- | --- | --- | --- | --- | --- |
| **AKI D1*** | 1 | 0.99 | 0.06 | 1.06 | 0.11 | 0.65 | 0.84 |
|  | 2 | 0.99 | 0.10 | 1.10 | 0.10 | 0.66 | 0.85 |
|  | 3 | 0.96 | 0.26 | 1.30 | 0.16 | 0.69 | 0.78 |
|  | 4 | 0.93 | 0.38 | 1.49 | 0.20 | 0.72 | 0.74 |
|  | 5 | 0.88 | 0.53 | 1.89 | 0.22 | 0.77 | 0.72 |
|  | 6 | 0.83 | 0.66 | 2.42 | 0.26 | 0.81 | 0.45 |
|  | 7 | 0.75 | 0.76 | 3.10 | 0.33 | 0.84 | 0.64 |
|  | 8 | 0.67 | 0.81 | 3.59 | 0.40 | 0.86 | 0.59 |
|  | 9 | 0.58 | 0.87 | 4.42 | 0.49 | 0.88 | 0.54 |
|  | 10 | 0.50 | 0.91 | 5.45 | 0.55 | 0.90 | 0.51 |
|  | 11 | 0.42 | 0.93 | 6.13 | 0.62 | 0.91 | 0.48 |
|  | 12 | 0.35 | 0.95 | 7.68 | 0.68 | 0.93 | 0.46 |
|  | 13 | 0.28 | 0.96 | 7.77 | 0.75 | 0.93 | 0.44 |
|  | 14 | 0.22 | 0.98 | 9.45 | 0.80 | 0.94 | 0.42 |
|  | 15 | 0.16 | 0.99 | 14.29 | 0.85 | 0.96 | 0.40 |
|  | 16 | 0.12 | 0.99 | 18.69 | 0.89 | 0.97 | 0.39 |
|  | | | | | | | |
| **AKI after D1**** | 1 | 0.98 | 0.10 | 1.09 | 0.19 | 0.57 | 0.81 |
|  | 2 | 0.96 | 0.15 | 1.13 | 0.26 | 0.58 | 0.76 |
|  | 3 | 0.89 | 0.36 | 1.39 | 0.30 | 0.63 | 0.73 |
|  | 4 | 0.82 | 0.50 | 1.66 | 0.35 | 0.67 | 0.70 |
|  | 5 | 0.75 | 0.66 | 2.18 | 0.38 | 0.73 | 0.69 |
|  | 6 | 0.63 | 0.77 | 2.77 | 0.48 | 0.77 | 0.63 |
|  | 7 | 0.50 | 0.83 | 3.04 | 0.60 | 0.79 | 0.58 |
|  | 8 | 0.39 | 0.92 | 4.95 | 0.66 | 0.86 | 0.56 |
|  | 9 | 0.32 | 0.96 | 8.15 | 0.71 | 0.91 | 0.54 |
|  | 10 | 0.20 | 0.97 | 7.83 | 0.82 | 0.91 | 0.50 |
|  | 11 | 0.14 | 0.98 | 8.04 | 0.88 | 0.91 | 0.48 |
|  | 12 | 0.11 | 0.99 | 8.24 | 0.90 | 0.91 | 0.48 |

Pre-test probability (or prevalence) for MAKE outcome = 0.634 and 0.548 in AKI D1 and AKI after D1, respectively

^!^+LR; positive likelihood ratio

^!!^-LR; negative likelihood ratio

^#^PPV; positive predictive value is numerically equal to positive post-test probability which calculated from positive likelihood ratio by using Fagan nomogram

^$^NPV; negative predictive value is numerically equal to 1 minus negative post-test probability which calculated from negative likelihood ratio by using Fagan nomogram

*AKI D1; AKI on the first day of ICU admission (n = 2,154) assess the score at 24^th^ hour

**AKI after D1; AKI after the first day of ICU admission (n = 702) assess the score at 48^th^ hour (please see detail of the reason why use at 48^th^ hour parameters in Supplement Table 3)

**Table S3. Discrimination comparison of SEA-MAKE score in patients with AKI-after-the-first-day (AKI after D1) assessing at 24^th^, 48^th^, and 72^th^ hour of admission (n = 702)**

|  | **AUC** | **Std. Error** | **95 % CI** | **Missing data, n** |
| --- | --- | --- | --- | --- |
| 24^th^ hour | 0.73 | 0.02 | 0.69, 0.77 | 0 |
| 48^th^ hour | 0.76 | 0.02 | 0.72, 0.80 | 190 |
| 72^th^ hour | 0.75 | 0.02 | 0.71, 0.80 | 248 |

Therefore, we proposed to use parameter at 48^th^ hour in assessing SEA-MAKE score in patient with AKI after D1 because of its highest AUC value.

**Table S4.** **Predictive ability of SEA-MAKE score in different setting**

| **Level** | **Number of Centers** | **Number of Patients** | **AUC** | **Std.Error** | **95 % CI** |
| --- | --- | --- | --- | --- | --- |
| - University* | 8 | 1,300 | 0.80 | 0.01 | 0.78, 0.83 |
| - Regional** | 5 | 1,055 | 0.81 | 0.01 | 0.78, 0.83 |
| - Provincial*** | 4 | 501 | 0.76 | 0.02 | 0.71, 0.80 |
| Total | 17 | 2,856 | 0.80 | 0.01 | 0.78, 0.81 |

*University hospitals provide medical services at the super tertiary care level.

**Regional hospitals have a capacity of at least 500 beds, capable of tertiary care level

***Provincial or general hospitals have a capacity of 200 to 500 beds, capable of secondary care level.

**Table S5. Discrimination of SEA-MAKE score for composite MAKE and each separated endpoints within 28 days after AKI**

| **Endpoints** | **AUC (95 % CI)**^#^ | **Number of Events**^#^ |
| --- | --- | --- |
| - MAKE (composite) | 0.80 (0.78, 0.81) | 1,750 |
| - Death | 0.72 (0.70, 0.74) | 1,184 |
| - New RRT | 0.80 (0.77, 0.81) | 410 |
| - Renal non-recovery | 0.76 (0.74, 0.77) | 1,163 |

^#^Data analyzed after 10 imputations

MAKE; Major Adverse Kidney Events, RRT; Renal Replacement Therapy


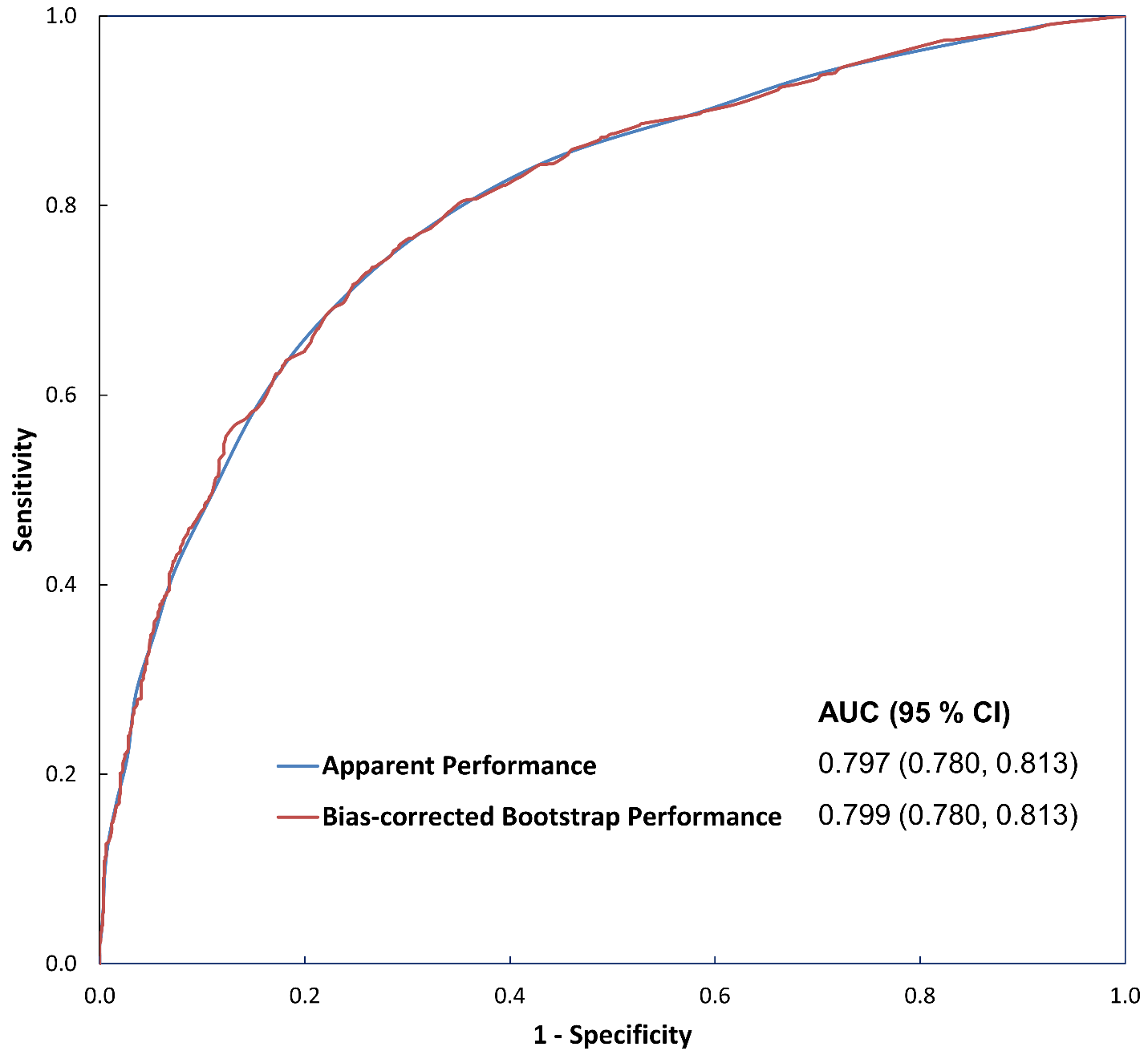


**Figure S1.** **The area under the receiver operating characteristic (ROC) curve for MAKE prediction of the apparent performance and bias-corrected bootstrap performance**


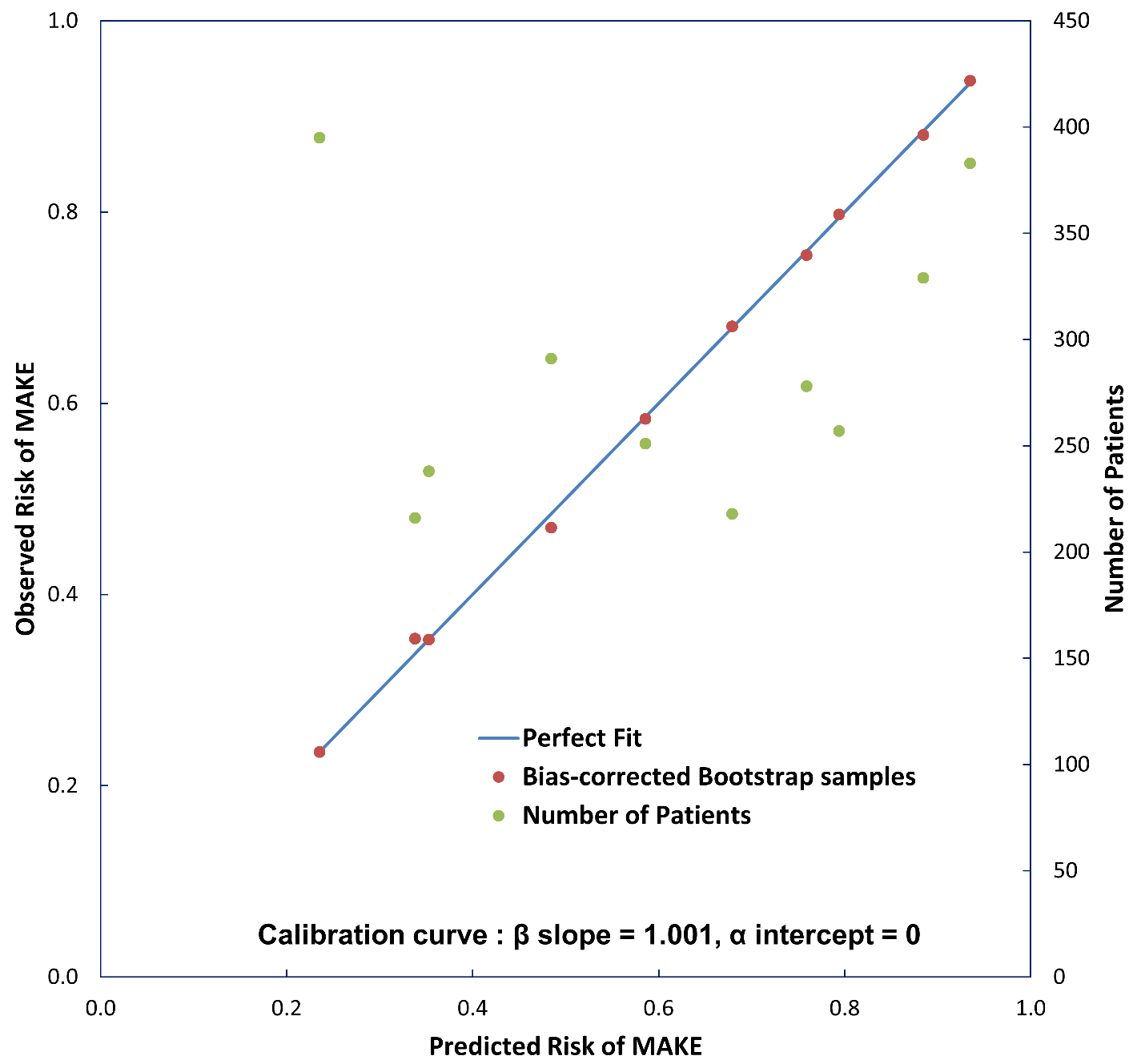


**Figure S2.** **Calibration curve analysis comparing between observed and predicted risk of MAKE at each cut-point of SEA-MAKE score in internal validation**

**
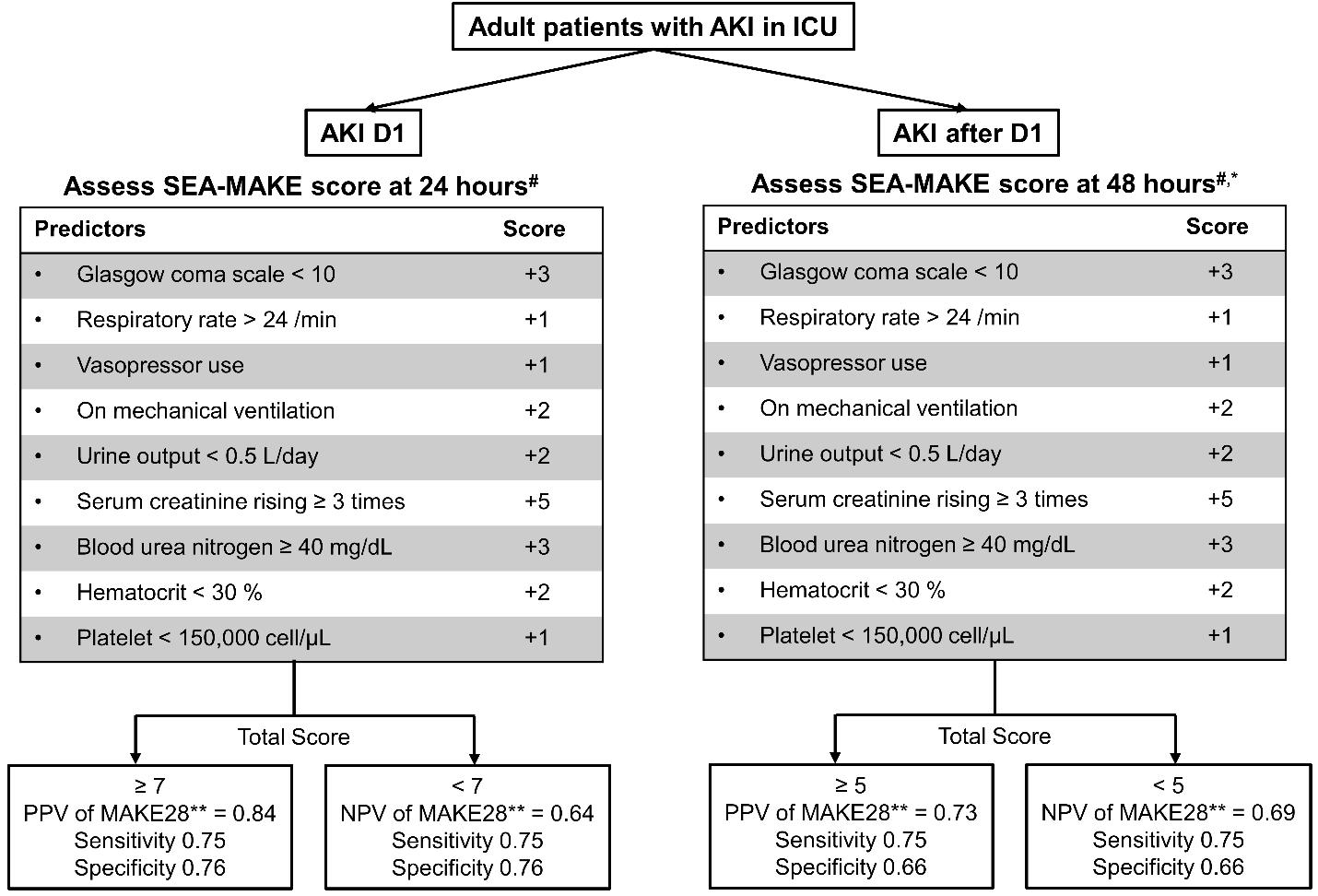
**

**Figure S3. The proposed diagram of SEA-MAKE score in clinical practice at the point of care**

^#^SEA-MAKE score uses the worst values of the predictors measured at 24th hour (AKI D1) and 48th hour (AKI after D1) of admission to the ICU

*Please see Supplement Table 4 for reason why we proposed to use 48th hour parameters in AKI after D1

**MAKE28; major adverse kidney events that occur in hospital within 28 days after AKI in ICU patients

PPV; positive predictive value

NPV: negative predictive value

**Table S6. TRIPOD Checklist**

| Section/Topic | Itemm |  | Checklist Item |  | Page |
| --- | --- | --- | --- | --- | --- |
| Title and abstract | | | Full examples of TRIPOD | This study (SEA-MAKE Score) |  |
| Title | 1 | D;V | Identify the study as developing and/or validating a multivariable prediction model, the target population, and the outcome to be predicted. | SEA-MAKE score as a tool for predicting Major Adverse Kidney Events in Critically ill Patients | 1 |
| Abstract | 2 | D;V | Provide a summary of objectives, study design, setting, participants, sample size, predictors, outcome, statistical analysis, results, and conclusions. |  | 2 |
|  | | | | |  |
| Background and objectives | 3a | D;V | Explain the medical context (including whether diagnostic or prognostic) and rationale for developing or validating the multivariable prediction model, including references to existing models. | If MAKE can be practically predicted at the point of care, it can help identifying high risk AKI patients for possible targeted preventive therapy. | 5 |
|  | 3b | D;V | Specify the objectives, including whether the study describes the development or validation of the model or both. | to create a scoring system for predicting major adverse kidney events within 28 days (MAKE 28) after AKI in ICU patients | 6 |
| Methods | | | | |  |
| Source of data | 4a | D;V | Describe the study design or source of data (e.g., randomized trial, cohort, or registry data), separately for the development and validation data sets, if applicable. | a prospective multicenter observational cohort study | 6 |
|  | 4b | D;V | Specify the key study dates, including start of accrual; end of accrual; and, if applicable, end of follow-up. | from February 2013 to July 2015 were enrolled in the study | 6 |
| Participants | 5a | D;V | Specify key elements of the study setting (e.g., primary care, secondary care, general population) including number and location of centres. | conducted in 17 ICUs throughout 16 hospitals from various regions across Thailand country. The hospital study sites included university, regional, and provincial settings. Patients 15 years of age or older admitted to the participating ICUs | 6 |
|  | 5b | D;V | Describe eligibility criteria for participants. | Patients 15 years of age or older admitted to the participating ICUs from February 2013 to July 2015 were enrolled in the study. | 6 |
|  | 5c | D;V | Give details of treatments received, if relevant. |  | - |
| Outcome | 6a | D;V | Clearly define the outcome that is predicted by the prediction model, including how and when assessed. | The primary outcome was patients who met one or more criteria for Major Adverse Kidney Events within 28 days (MAKE28); hospital death, provision of dialysis, and a sustained loss of kidney function | 7 |
|  | 6b | D;V | Report any actions to blind assessment of the outcome to be predicted. |  | - |
| Predictors | 7a | D;V | Clearly define all predictors used in developing or validating the multivariable prediction model, including how and when they were measured. | The data were consecutively collected every day for the first 7 days and then weekly on day 14, 21, and 28. However, only first day parameters were used as predictors to create the model. | 7 |
|  | 7b | D;V | Report any actions to blind assessment of predictors for the outcome and other predictors. |  | - |
| Sample size | 8 | D;V | Explain how the study size was arrived at. | All available data was used to maximize the power and generalizability of the proposed model. | 6 |
| Missing data | 9 | D;V | Describe how missing data were handled (e.g., complete-case analysis, single imputation, multiple imputation) with details of any imputation method. | Candidate variables with more than 25 % missing data were eventually excluded. As data were not missing completely at random, multiple imputations (n=10) for the missing candidate variables were performed in the final model. | 10 |
| Statistical analysis methods | 10a | D | Describe how predictors were handled in the analyses. | Categorical data were presented as counts and percentages. Continuous data were presented as mean and standard deviation (SD) if normally distributed or median with interquartile range if non-normally distributed. | 9 |
|  | 10b | D | Specify type of model, all model-building procedures (including any predictor selection), and method for internal validation. | In multivariable analysis models, each clinical factor was adjusted for age, gender, body mass index, and co-morbidities including hypertension, diabetes, coronary artery disease, cerebrovascular disease, malignancy, and chronic kidney disease to examine the potential predictability. Then, significant predictors were included in final analysis. | 9 |
|  | 10c | V | For validation, describe how the predictions were calculated. | Internal validation was performed by using bootstrapping method[19]. A total of 2,000 bootstrap samples were drawn | 10 |
|  | 10d | D;V | Specify all measures used to assess model performance and, if relevant, to compare multiple models. | The apparent performance of the score was evaluated using the following performance measures: overall performance was evaluated by Nagelkerke’s R^2^; discrimination (ability to differentiate patients with MAKE outcome from patients with non-MAKE outcome) was evaluated using the area under the receiver operating characteristic curve (AUC), perfect discrimination would be described if an AUC =1 while discrimination no better than chance would be described if AUC = 0.5; and calibration (ability to generate predictions that are on average close to the average observed outcome) was evaluated by the calibration intercept α and slope β from the linear regression of the observed outcomes (dependent variable) vs. the predicted risks (independent variable) with visually using a calibration plot | 10 |
|  | 10e | V | Describe any model updating (e.g., recalibration) arising from the validation, if done. | - | - |
| Risk groups | 11 | D;V | Provide details on how risk groups were created, if done. | - | - |
| Development vs. validation | 12 | V | For validation, identify any differences from the development data in setting, eligibility criteria, outcome, and predictors. | - | - |
| Results | | | | |  |
| Participants | 13a | D;V | Describe the flow of participants through the study, including the number of participants with and without the outcome and, if applicable, a summary of the follow-up time. A diagram may be helpful. | All 5,381 cases were in this cohort. However, after exclusion (382 cases from unavailable blood and urine samples, and 258 cases from ESRD) 5,071 cases were in analysis (Figure 1). | 11 |
|  | 13b | D;V | Describe the characteristics of the participants (basic demographics, clinical features, available predictors), including the number of participants with missing data for predictors and outcome. | MAKE had mean age 65.8 years old which was not found to be significantly different with non-MAKE. While many parameters including diabetes, CKD, Glasgow Coma scale, body temperature, heart rate, respiratory rate, mean arterial pressure, hematocrit, platelet count, serum sodium, serum potassium, blood urea nitrogen, serum creatinine, total bilirubin, PaO_2_/FiO_2_ ratio, arterial pH, urine output, net fluid balance, mechanical ventilation, and vasopressor use | 12 |
|  | 13c | V | For validation, show a comparison with the development data of the distribution of important variables (demographics, predictors and outcome). | - | - |
| Model development | 14a | D | Specify the number of participants and outcome events in each analysis. | are shown in Table 1. | 12 |
|  | 14b | D | If done, report the unadjusted association between each candidate predictor and outcome. | univariate analysis for comparisons of clinical and laboratory characteristics between MAKE and non-MAKE at the time of enrollment are shown in Table 1. | 12 |
| Model specification | 15a | D | Present the full prediction model to allow predictions for individuals (i.e., all regression coefficients, and model intercept or baseline survival at a given time point). | Nine predictors were significant in the final model including low Glasgow coma scale OR (95 % CI) = 2.72 (2.17, 3.40), tachypnea OR (95 % CI) = 1.55 (1.29, 1.86), vasopressor use OR (95 % CI) = 1.56 (1.30, 1.88), on mechanical ventilation OR (95 % CI) = 1.81 (1.45, 2.27), oliguria OR (95 % CI) = 1.78 (1.48, 2.14), serum creatinine rising ≥ 3 times OR (95 % CI) = 4.77 (3.38, 6.73), high blood urea nitrogen OR (95 % CI) = 2.33 (1.89, 2.86), low hematocrit OR (95 % CI) = 1.81 (1.50, 2.19), and thrombocytopenia OR (95 % CI) = 1.40 (1.15, 1.70) (Table 2); | 13 |
|  | 15b | D | Explain how to the use the prediction model. | nine parameters that used in the score including Glasgow coma scale, respiratory rate, vasopressor use or not, on mechanical ventilation or not, urine output, serum creatinine rising, blood urea nitrogen, hematocrit, and platelet count that all using the worst values of the predictors measured within first 24 hours of admission to the ICU. | 15 |
| Model performance | 16 | D;V | Report performance measures (with CIs) for the prediction model. | Discrimination (AUC) of apparent performance was 0.797 (95 % CI: 0.780-0.813), bias-corrected bootstrap performance was 0.799 (95 % CI: 0.780-0.813) (Supplement Figure 1), and optimism-corrected performance was 0.795. | 13 |
| Model-updating | 17 | V | If done, report the results from any model updating (i.e., model specification, model performance). | - | - |
|  | | | | |  |
| Limitations | 18 | D;V | Discuss any limitations of the study (such as nonrepresentative sample, few events per predictor, missing data). | However, several limitations which need to be discussed in this study. First, the diversity of the patients in study population could be problematic because specific diseases especially the cause of AKI may affect clinical courses and outcomes but we could not include it in this score. Second, laboratory parameters that were used in creating SEA-MAKE score were measured and reported at study sites, so adequate optimizing for standardization of laboratory test results among the specific study sites were another important issue to be concerned. | 17 |
| Interpretation | 19a | V | For validation, discuss the results with reference to performance in the development data, and any other validation data. | In summary, the nine parameters that used in the score including Glasgow coma scale, respiratory rate, vasopressor use or not, on mechanical ventilation or not, urine output, serum creatinine rising, blood urea nitrogen, hematocrit, and platelet count that all using the worst values of the predictors measured within first 24 hours of admission to the ICU. These predictors are routinely measured and recorded in ICU care. | 15 |
|  | 19b | D;V | Give an overall interpretation of the results, considering objectives, limitations, results from similar studies, and other relevant evidence. | The performance of SEA-MAKE score and the McKown AC et al’s model were shown in Supplement Table 3, however, the comparison is not only difficult because of the differences in study populations, event-rates and case-mix, but also the comparisons should perfectly be head-to-head comparisons in the same external cohort. | 15 |
| Implications | 20 | D;V | Discuss the potential clinical use of the model and implications for future research. | These predictors are routinely measured and recorded in ICU care. Importantly, the score is easy to calculate and can be applied to most hospitals even in resource limited settings. The Supplement Figure 3 showed flowchart of how to use SEA-MAKE score at the point of care. | 15 |
| Other information | | | | |  |
| Supplementary information | 21 | D;V | Provide information about the availability of supplementary resources, such as study protocol, Web calculator, and data sets. | On reasonable request, data from this study will be available from the corresponding author. | 18 |
| Funding | 22 | D;V | Give the source of funding and the role of the funders for the present study. | Financial support for the study was provided by International Society of Nephrology (ISN), The Kidney Foundation of Thailand, and the Medical Association of Thailand. | 19 |

*Items relevant only to the development of a prediction model are denoted by D, items relating solely to a validation of a prediction model are denoted by V, and items relating to both are denoted D;V. We recommend using the TRIPOD Checklist in conjunction with the TRIPOD Explanation and Elaboration document.
